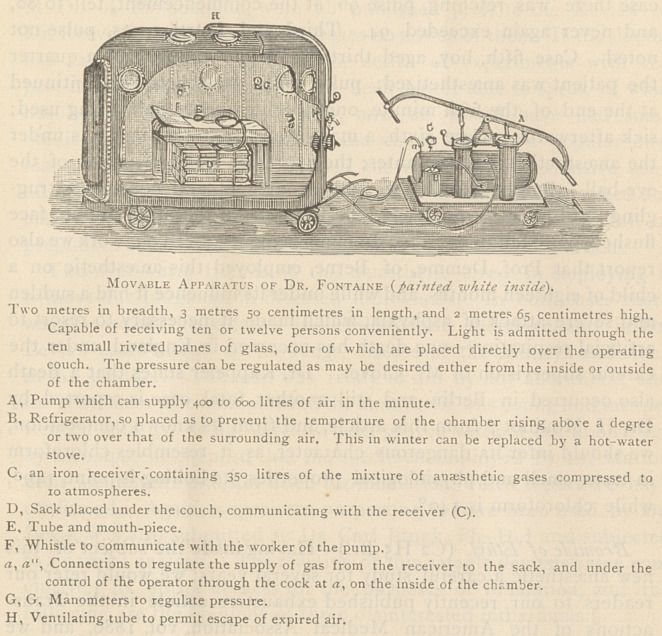# Nitrous Oxide Gas

**Published:** 1881-08

**Authors:** Laurence Turnbull

**Affiliations:** Philadelphia


ARTICLE IV.

Continued from page 391.

### NITROUS OXIDE GAS.

BY DR. L. TURNBULL, PHILADELPHIA.

The practical application of the principles of M. Paul Bert and his apparatus are as follows: "The first operation under pressure was performed by M. P. Bert's method Feb. 13, 1879. M. Leon Labbe operated in a chamber of compressed air (total pressure 0.92) on a patient for inturned toe-nail, with a mixture of nitrous oxide gas (85 pts.) and oxygen (15 pts.). In fifteen seconds from the first inhalation, without any unpleasant effects of the pure gas, insensibility and muscular relaxation were complete; speedy return to sensibility followed. M. Pean set to work to apply the method of Paul Bert in the domain of practical surgery. His first operation was the removal of the breast. The operation lasted fourteen minutes, and the patient respired 150 litres of a mixture of nitrous oxide gas and hydrogen. The effect was perfect; the patient without assistance got off the operating couch. Following up the experience of this operation, M. Pean has constructed a chamber (movable) for the administration of the gas under pressure, varying from 15 to 22 centimetres, rarely pushed to 26; and with such an apparatus M. Pean, at the Hospital St. Louis, and M. Labbe at the Lareboisere. have performed a great number of operations. Dr. Fontaine, of the Rue Chateaudun, having brought his special knowledge to bear in constructing the following ingenious but simple pneumatic cylinder, (Fig. 1) in which the administration is conducted.

### BICHLORIDE OF METHYLENE.

*The bichloride of methylene* we mentioned in our work and reported seven deaths; this has been confirmed by Dr. Kappeler, who adds two more deaths to the list; and experiments in this city, where we had it made upon ourselves, and by the late Dr. Atlee, confirm its dangerous character, with no particular advantages in ovariotomy, except in the hands of an English operator.

*Ethidene Dichloride.*—We have given a report of six cases in which this anæsthetic was employed in January, 1879. In the first case the pulse went up from 98 to 120, full and bounding; in the fourth minute it fell to 98, and the eighth to 76. The anæsthetic was discontinued, one drachm and a half having been used. In the second case there was retching, pulse 96 at the commencement, fell to 80, and never again exceeded 94. Third and fourth cases, pulse not noted. Case fifth, boy, aged thirteen, in one minute and a quarter the patient was anæsthetized; pulse 108. Anæsthetic discontinued at the end of the fifth minute, one drachm and a half being used; sick afterwards. Case sixth, a man, aged twenty; patient was under the anæsthetic sixteen minutes; the operation was extirpation of the eye-ball. Pulse reached 110, with excitement and conscious struggling until he was completely anæsthetized; in the ninth minute face flushed, pulse fell to 92. Five drachms being used. In our work we also report that Prof. Demme, of Berne, employed this anæsthetic on a child of eighteen months, and while under its influence it had a sudden and severe attack of asphyxia, which made it necessary to resort to artificial respiration—one death has occurred in England under the careful supervision of Mr. Clover. Dr. Kappeler states that a death also occurred in Berlin, and still another fatal case is reported by Dr. H. Mouillot ; from this article, and from its known composition, we should infer its dangerous character, as it resembles chloroform in taste, smell and physiological properties, its boiling is point 149°, while chloroform is 140°.

*Bromide of Ethyl.* ($C_2 H_5 Br.$)—Having made the subject of this new anæsthetic a careful study for several years; we would refer our readers to our recently published exhaustive article in the Transactions of the American Medical Association, vol. 1880, and we shall only give here a short abstract. Bromide of Ethyl or Hydrobromic ether has been now in use since 1876–8 in some 2,000 cases, and we were the first to employ it, and upon ourselves, in this country, having been brought completely under its influence, at least on six different occasions, in no instance did it increase the pulse over 120 ; in no instance did it oppress or distress respiration ; the sleep was calm, and on awaking we were not even in the slightest nauseated. We gave ether to reptiles, birds, cats and dogs, and made post-mortems, and unless we wished the animal to die, we had them completely under our control. The recent experiments by us before the Pennsylvania Dental College, published extensively,* or those of Dr. Ott, of Easton, Pennsylvania, or Prof. H. C. Wood,

*Independent Practitioner & Med. & Surg. Reporter, 1880.

M. D., of Philadelphia, were all published prior to the Review of Dr. Reeve, in July, 1880, but he takes no notice of them. If the doctor had taken record of each of the series of experiments with the post-mortem, etc., he would have been better able to speak of what has been done in this country, and we would have had some credit abroad.* When the new anæsthetic became a popular one, many came forward and claimed the credit by its extensive use; ultimately the influence of it on man and animals was tested far and wide by some who were honest, and others who were full of prejudices because they did not introduce it or make the article.

Two over-warm friends of this anæsthetic erred in employing it without due judgment in diseased individuals. In both cases there was disease of the kidneys and epilepsy and diarrhœa, and in Dr. Levis's cases disease of the lungs, disease of the kidneys and stone in the bladder. The man was ready to die by any anæsthetic, and the gentleman who administered the anæsthetic was young and inexperienced with it. It had already been proven without our knowledge by a competent chemist, that the specimen employed by Dr. Marion Sims was impure and not fitted for inhalation, which report we have confirmed. Again, a specimen purporting to have been used by Dr. Levis, was also submitted to Dr. Carl Jungk, Ph. D.,† and subjected to a careful analysis. The reaction of this sample demonstratee conclusively its entire ufitness for the purposes of inhalation, and the following are the opinions of three disinterested individuals:‡

APPLICATION AND ACTION OF BROMIDE OF ETHYL AND ITS INFLUENCE UPON THE GROWTH AND GERMINATION OF PLANTS.

After a short introduction the author says as follows:

1. "Bromide of Ethyl always proved itself a very efficient anæsthetic, and was used with good results, both by myself and other physicians, both in France and America. Further experiments overcame the few doubts that existed about its anæsthetic properties."

2. "After using Bromide of Ethyl internally myself, I applied it

* See our full report of all the experiments made in a volume of Transactions for 1879–80, of the American Medical Association.

†Therapeutic Gazette, Detroit, 1880.

‡ Rabuteau in Gazette Medicale de Paris, August, 1880, No. 32.

in two cases of inflammation of the stomach. Both times I had good results. I found that it had neither a caustic nor an irritant effect on the mucuous membrane. It stopped all pain at once and did not diminish the appetite in the least, but, on the contrary, increased it. The latter result was not at all surprising to me, since Trousseau had made the observation that common ether increases the appetite."

1. "I applied vapor of Bromide of Ethyl in cases of whooping cough, measles, and common cough, with favorable results. In the last case, I tried it personally, and found that it gave immediate relief. Taken all in all, Bromide of Ethyl is a valuable anæsthetic and sedative. It produces anæsthesia far more rapidly than chloroform; and the patient recovers more quickly than after the use of chloroform; at least, according to my experience with animals."

I found it very valuable in stomachic cramp with irritation; in ear-ache, when introduced into the ear on cotton wool, it immediately relieves the pain. Of course, chemical purity is essential.

Besides this, the author announces that he made experiments with vapor of Bromide of Ethyl, such as Claude Bernard with chloroform, to notice its effect upon the growth and germination of plants, which are very interesting. It has no great hindering influence upon the vegetation."

BROMIDE OF ETHYL AND ANÆSTHESIA.*

"Many were inclined to believe that Drs. Turnbull and Levis had allowed their zeal to bias their judgment in regard to bromide of ethyl, and that through an infirmity of mind, to which humanity is but too liable, their prepossession blinded them to the inherent defects of the drug. Others knew that such suspicions were unjust, and attributed the train of disastrous results to the impurities in the preparations employed. The gentlemen who have founded their unequivocal endorsement of the superiority of bromide of ethyl on experience in its use in over 200 (over 1,000 by other experiments, if we can judge by the amount sold) cases, were, doubtless, very much surprised at the results of its use in other hands; and it was very natural for them, and those who had confidence in their integrity, to attribute the unfavorable experience rather to impurities

---

***The Therapeutic Gazette*, Detroit, Sept. 10, 1880.

in the samples employed than to any inherently deleterious properties in the drug itself. The analysis given by us in our June number of nine samples selected from the products of the different manufacturers certainly goes very strongly to support this view.

In the light of the experience thus far had with bromide of ethyl the following conclusions seem clearly deducible :

It is an anæsthetic of pronounced strength, pleasant of administration, speedy in its action, and very speedily eliminated, the patient recovering from its effects very readily. As regards its safety, the use of the pure drug is not more apt to be followed by fatal results than is that of the other anæsthetics usually employed. It is not safer, but is more pleasant than sulphuric ether ; it is not more pleasant, but is safer than chloroform.

Any agent capable of producing anæsthesia cannot be too carefully administered, and, probably, the careless and lavish manner in which it is usual to administer ether led to the same abuse of bromide of ethyl, possibly the unfavorable effects of the later anæthestic are traceable to this cause, its pleasant odor tempting to this abuse. The method of administration recommended by Dr. Levis is to take a folded napkin large enough to cover the entire face, in the centre of which are to be pinned several folds of soft linen about four inches square. The anæsthetic is to be poured on this linen. The patient, being upon his back, is instructed to breathe quietly and without effort, and when he has been tranquilized the bromide of ethyl is applied promptly and at such intervals and in such quantities as will ensure a constant supply of the vapor, the object being to bring him as speedily as possible under its influence. The latter point seems important, in this respect bromide of ethyl being similar to sulphuric ether, and differing from chloroform, in the administration of which a free admixture of atmospheric air is generally considered essential to safety.

Notwithstanding the reported cases of excessive vomiting from the use of bromide of ethyl, the most reliable evidence goes to show that with the pure drug properly administered vomiting rarely, if ever, occurs. As a precaution, however, an empty condition of the stomach should be insisted on, and no food of a solid nature should be partaken of for at least four hours before the giving of the anæsthetic.

The same care should be exercised in selecting the subject for anæsthesia by bromide of ethyl, as is, or should be, taken in the case of chloroform. It is positively known that the two fatal cases from its use which are reported, there existed defects in vital organs, which were very important factors in the fatal result.

The conclusion, from its history, is, that bromide of ethyl, although not justifying the hope as first entertained of it, that it would combine agreeableness of administration with absolute safety, is, nevertheless, a valuable addition to its class of remedies. It seems, however, to be more liable to contain deleterious ingredients, the result of changes taking place in the process of its manufacture than are other anæsthetics, and the necessity that the sample employed shall be chemically pure, is imperative."

Samples from both the above manufactures referred to, we obtained and only numbered them, and then submitted them to analysis by Prof. Barker, of the University of Pennsylvania, when he found them to contain dangerous impurities.

It is one of the saddest phases of the present age that men, honest in other matters, will manufacture and sell impure chemicals and pharmaceutical agents, regardless of the potent influence for evil they may have upon the community, while if they administered a poison to an individual knowing it to be such they would be tried and found guilty of murder, and would have to suffer the penalty, and yet they do the same thing under the guise of commercial specimens of poisonous narcotics. There ought to be a proper officer appointed by the government to guard against adulterations and sophistication; as there is not, we physicians are often at fault in not specifying the manufacturer's name on the article we have found pure and reliable, and *vice versa.* We know, as a graduate of the Philadelphia College of Pharmacy, that it is not either agreeable or creditable to the art of Pharmacy that a physician is obliged to do this thing, but it is, unfortunately, the case, that the druggist's interest is chiefly commercial, and he is tempted to buy from whom he can obtain the cheapest, but not the purest.

### LOCAL ANÆSTHESIA.

The application of the spray of ordinary ether is employed in cases of abscess, fistula, hemorrhoids, etc. Some conditions (Rottenstein) are considered indispensable; the ether should be of a specific gravity not exceeding 0.723; it should boil in the palm of the hand; placed on the tongue, it should evaporate rapidly—directed in a jet on the bulb of a thermometer it should lower the mercury to six degrees below zero, and produce snow by the condensation of the air; on the back of the hand it should produce a slight deposit of white frost, followed by a general pallor of the skin and complete insensibility." One of the great drawbacks to its use in this form is the pain it produces in freezing.

In neuralgia, and in certain operations, M. Adrian uses chloro-carbon differing from chloroform in composition by one equivalent of chloride. It is less stimulating and irritating than chloroform. It can be administered internally, suspended in oil of sweet almonds and gum arabic (Vee) and appliedexternally in the following formula: Axungia, 20 grammes; cera alba, 4 grammes; chloro-carbon, 6-8-12 grammes.

Dr. Vigoroux considers that the power of the induced current for electrical anæsthesia is increased in persons who are readily influenced by metals (metallotherapy), magnetism, galvanism, mesmerism, etc., in short, the hysterical temperament.

HYDROBROMIC ETHER, OR THE BROMIDE OF ETHYL, AS A LOCAL ANÆSTHETIC.

We have employed this agent in numerous instances for obtaining local anæsthesia, with very little pain, and with great satisfaction in from one to two minutes. When a tumor or abscess is situated below the skin, it is then necessary to continue the spray a longer period. In a recent operation at the angle of the jaw, in which a cyst was removed, the first deep cut of the knife gave no pain, and by keeping the spray around and near the edge of the swelling so as not to blanche the skin, it had the desired effect of relieving the acute pain when dissecting out the sack of the piogenic membrane. The spray apparatus was of large caliber (Lefferts), which acts much better than the ordinary "Richardson," which I found gave too fine a spray. This ether has great advantages over ordinary ether in its non-liability to ignition when employing the thero-cautery.

COMBINATIONS OF ANÆSTHETICS.

*Chloral and Chloroform combined.*—"Forné" stated that the effects were the same as when morphia and chloroform were combined—preventing excitation and increasing the effect. "Dolbeau and Demarquay" think it dangeous. The combination is uncertain and dangerous, whether given by the mouth or rectum.

*Morphia and Chloroform.*—"Poncet and Chauvel" have relinquished its use in the military service of France.

*Morphia and Ether.*—Vomiting, according to the experience of Kappler, occurred in nine out of twenty-five administrations; twelve failed.

*Chloral and Ether.*—Though better than the combination of morphia and ether, it has no special advantages over ether alone.

Notwithstanding the enormous advantages of ether, notwithstanding its supremacy, we do not hesitate to recommend chloroform under the exceptional circumstances in which the ambulance surgeon is surrounded (Rottenstein). The opinions of M. Baudens, 1847, )in the case of military conscripts) Boussou, 1847, (Gazette Medicale, 34 -37); Bayard, (Annales d'Hygene et de Medicine Legale,); Martino, Sedillot, MM. Briand and Chaude (Traite de Medicine Legale); MM. Champouillon and Devillers are quoted by the same authority in discussing the use of anæsthetics, in detecting imposition, the utility of the same in detecting assumed muscular contractions, paralysis, feigned madness and imbecility, aphonia. (See Turnbull's "Manual of Diseases of the Ear, p. 212). Patients during or recovering from anæsthesia will speak loudly and sing, who previously were both deaf and dumb.

We have already referred to the risk run by the physician, surgeon or dentist in administering anæsthetics, especially chloroform to females, not only of criminal charges, which have been made in courts of justice, but of deaths by the hands of the supposed injured husband. Dr. Rottenstein gives in full the discussion that occurred at the French Medico-Legal Congress—M. Devillers, president. The sensibility of the erectile tissues and of the genital organs persists after it is lost in other portious of the body; and hence, is shown by Lietaud, the tendency there is to voluptuous dreams in woman, in consequence of the extreme sensitiveness of the vagina during the anæsthetic state. Hence M. Lacassagne insisted on the necessity of having two physicians in administering anæsthetics. M. Comby also spoke of the criminal liability whlch may ensue as a consequence of these delusions on the part of young females. M. Gallard strongly urged the risk which may follow the administration of anæsthetics if only one person be present.

## Figures and Tables

**Figure f1:**